# Neutrophils can promote clotting via FXI and impact clot structure via neutrophil extracellular traps in a distinctive manner in vitro

**DOI:** 10.1038/s41598-021-81268-7

**Published:** 2021-01-18

**Authors:** Y. Shi, J. S. Gauer, S. R. Baker, H. Philippou, S. D. Connell, R. A. S. Ariëns

**Affiliations:** 1 LIGHT Laboratories, Discovery and Translational Science Department, Institute of Cardiovascular and Metabolic Medicine, University of Leeds, Clarendon Way, Leeds, LS2 9LU UK; 2 Department of Physics, Wake Forest University, Winston Salem, NC USA; 3 The Astbury Centre for Structural Molecular Biology, Molecular & Nanoscale Physics, University of Leeds, Leeds, LS2 9JT UK

**Keywords:** Cell biology, Cardiovascular biology

## Abstract

Neutrophils and neutrophil extracellular traps (NETs) have been shown to be involved in coagulation. However, the interactions between neutrophils or NETs and fibrin(ogen) in clots, and the mechanisms behind these interactions are not yet fully understood. In this in vitro study, the role of neutrophils or NETs on clot structure, formation and dissolution was studied with a combination of confocal microscopy, turbidity and permeation experiments. Factor (F)XII, FXI and FVII-deficient plasmas were used to investigate which factors may be involved in the procoagulant effects. We found both neutrophils and NETs promote clotting in plasma without the addition of other coagulation triggers, but not in purified fibrinogen, indicating that other factors mediate the interaction. The procoagulant effects of neutrophils and NETs were also observed in FXII- and FVII-deficient plasma. In FXI-deficient plasma, only the procoagulant effects of NETs were observed, but not of neutrophils. NETs increased the density of clots, particularly in the vicinity of the NETs, while neutrophils-induced clots were less stable and more porous. In conclusion, NETs accelerate clotting and contribute to the formation of a denser, more lysis resistant clot architecture. Neutrophils, or their released mediators, may induce clotting in a different manner to NETs, mediated by FXI.

## Introduction

Neutrophils, which are produced in the bone marrow, are the most abundant class of leukocyte^1,2^. Neutrophils are known to participate in the innate inflammatory system as the earliest line of defense against microbial infection^3,4^. Recently, there has been increasing evidence that blood coagulation is associated with neutrophils^5–7^, including evidence that neutrophils contribute to thrombosis by interacting with both the injured endothelium and fibrin(ogen)^8–10^. Neutrophils can also influence thrombosis through the release of a number of active proteases and other agents such as matrix metalloproteinases (MMP), serine proteases (e.g., cathepsin G and elastase) and platelet-activating factor (PAF)^5,11^. For instance, cathepsin G and elastase are known to activate platelets^12–14^, factor V, VIII and X^15–17^. Moreover, MMP, cathepsin G and elastase cleave and inactivate tissue factor pathway inhibitor (TFPI)^18,19^ and elastase degrades antithrombin III^20^.

A key function via which neutrophils contribute to innate immune responses and also to thrombosis is the generation of neutrophil extracellular traps (NETs), which are released by activated neutrophils, and contain DNA and granular proteins (histone 3)^21,22^. There are various stimuli that can induce the generation of NETs (NETosis), such as bacteria, activated platelets and Phorbol 12-myristate 13-acetate (PMA)^23,24^. NETosis is a special type of cell death process that differs from apoptosis and necrosis, which enables neutrophils to retain their antibacterial function after the end of their lives^25^. It is widely known that NETs have properties of trapping and killing bacteria and pathogens^26,27^. More recently, NETs have also been shown to be involved in the regulation of procoagulant factors (e.g. Factor XII and platelet and von Willebrand factor (vWF))^22,28^. Thereby, NETs are able to provide a scaffold for the aggregation and adhesion of platelets and red blood cells, which may result in increased stability of clots^29,30^. This scaffold can be dismantled by the anticoagulant heparin and DNAses^30,31^. Histones are able to recruit fibrinogen and vWF, which can trigger platelet aggregation^30,32^, and can also induce platelet aggregation directly^30,32^. Histones also interact with fibrinogen and contribute to the heparin-induced platelet aggregation, a process enhanced by their regulation of αIIbβ3 activation, the integrin receptor on platelets^32^.

Although previous studies have shown that neutrophils and NETs are involved in both arterial and venous thrombosis^33–37^, questions remain whether neutrophils contribute to coagulation independently or via NETs, whether both neutrophils and NETs promote clotting via the intrinsic pathway and how procoagulant factors interact with neutrophils or NETs. This in vitro study aims to investigate the role of neutrophils and NETs in blood coagulation, fibrin formation, clot structure/density and clot stability, to decipher how neutrophils or NETs interact with the fibrin network. The role of neutrophils or NETs on clot formation and clot dissolution was analysed by turbidity and lysis assays, clot structure was determined by confocal microscopy, and clot stability and pore size were investigated using permeation studies. We found that both partially activated neutrophils and NETs promote clotting in plasma independently of additional triggers. We also found that the procoagulant effects of NETs are independent of FXI, FXII or FVII. However, the procoagulant effects of neutrophils are mediated via FXI, to a lesser extent by FXII, but not by FVII.

## Results

### Neutrophil-surface antigens on neutrophils and PLB-985 cells

**Figure 1:**
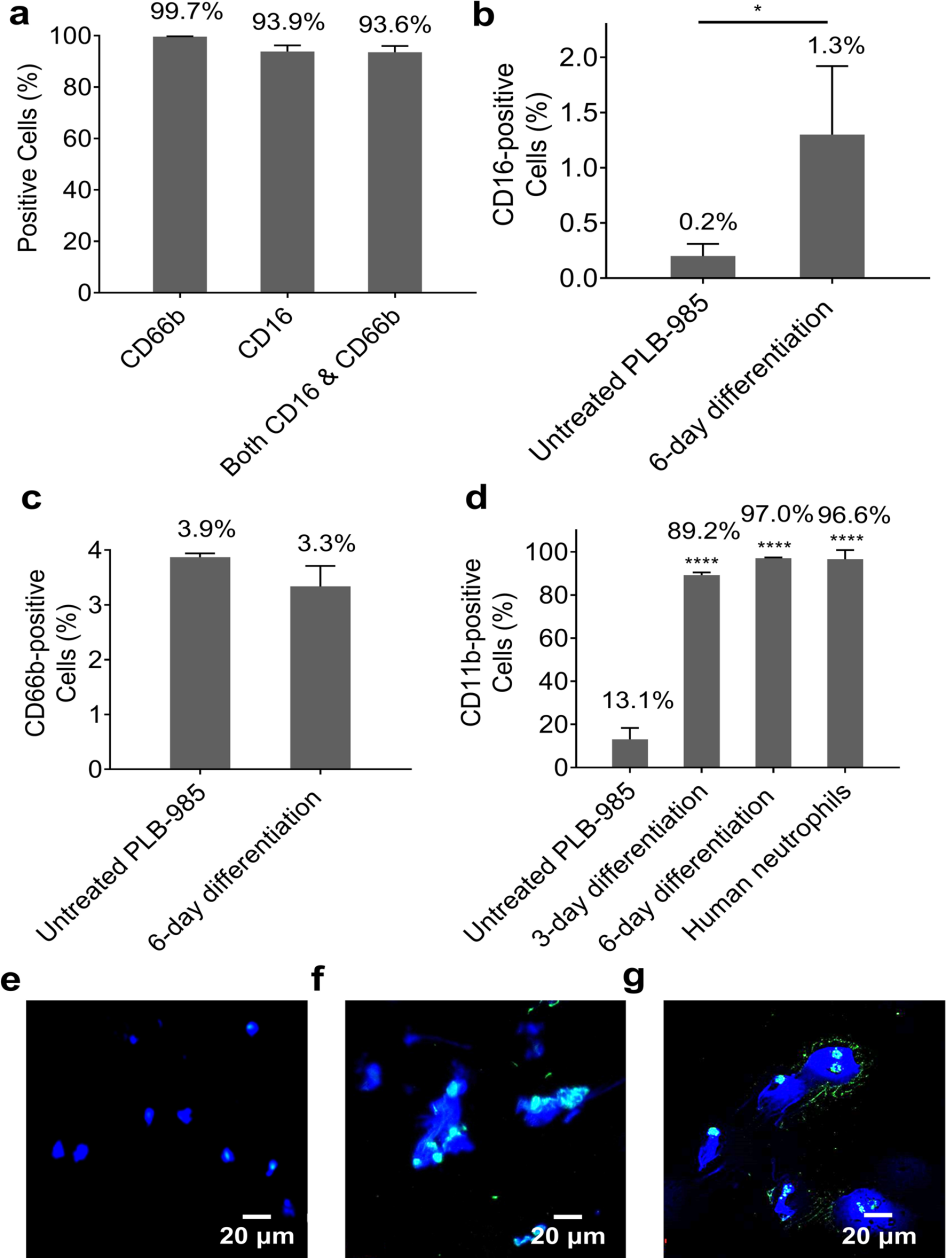
Expression of neutrophil-surface antigens on isolated human neutrophils and differentiated PLB-985 cells and the capacity of NETosis of these cells. The expression of antigens on cells was detected in living cells via flow cytometry. (**a**) Expression levels of CD16 and CD66b on human neutrophils were used as the positive control. Expression levels of (**b**) CD16 and (**c**) CD66b on differentiated PLB-985 cells were detected on the day 6 of treatment with 1.25% DMSO, untreated cells were used as the negative control. (**d**) Expression level of CD11b on differentiated PLB-985 cells was detected on the day 3 and day 6 of treatment with 1.25% DMSO, untreated PLB-985 cells were used as a negative control, and human neutrophils were used as a positive control. Error bars represent SD of three technical replicates. *P < 0.05, ****P < 0.0001. (**e**) Unstimulated human neutrophils using fluorescence microscopy. (**f**) NETs generated from isolated human neutrophils stimluated with PMA. (**g**) NETs generated from differentiated PLB-985 cells stimulated with PMA. Blue: DAPI-stained DNA. Green (cyan in overlay with blue): Alexa Fluor 488 labelled Histone H3 (citrulline R2 + R8 + R17).

To determine the purity of isolated human neutrophils, flow cytometry was carried out to analyse the expression of human neutrophil surface markers CD66b and CD16 on isolated human neutrophils. After isolation, 99.7% of cells expressed CD66b, 93.9% of cells expressed CD16, and 93.6% of cells expressed both (Fig. 1a). To identify whether PLB-985 cells differentiated into a functional neutrophil-like phenotype, the expression of CD16 and CD66b on differentiated PLB-985 cells was investigated via flow cytometry on day 6 of treatment, with untreated PLB-985 cells as the negative control. The expression of CD16 on differentiated cells (1.3%, *P* = 0.0389) was significantly higher than that on untreated cells (0.2%) (Fig. 1b). However, both CD16 and Cd66b (3.3%) had a very low expression level on differentiated PLB-985 cells (Fig. 1b,c). In order to find a positive marker for the differentiation of PLB-985 cells, the expression of another human neutrophil surface marker CD11b on differentiated cells was investigated by flow cytometry. Surface markers were detected on day 3 and day 6 of differentiation, isolated human neutrophils were used as positive control and untreated PLB-985 cells were used as negative control. As shown in Fig. 1d, differentiated PLB-985 cells had significantly increased expression level of CD11b (*P* < 0.0001) after treatment, as compared with untreated cells (13.1%). Namely, 89.2% of cells expressed CD11b on day 3 of differentiation and 97.0% of cells expressed CD11b on day 6 of differentiation. As days of treatment increased so did the expression level of CD11b on differentiated PLB-985 cells. The expression levels of CD11b on differentiated PLB-985 cells on day 6 of differentiation was similar to isolated blood neutrophils (96.6%).

### Visualization of NETs

Immunofluorescence was carried out to visualize NETs which were generated from isolated human neutrophils and differentiated PLB-985 cells. To better observe NETs, we stained samples for DNA using the blue dye 4′,6-diamidino-2-phenylindole (DAPI) and labeled them green with Alexa Fluor 488 labelled Histone H3 (citrulline R2 + R8 + R17), a marker for NETosis. Human neutrophils not stimulated with Phorbol 12-myristate 13-acetate (PMA) were used as negative control (Fig. 1e). Both human neutrophils and differentiated PLB-985 cells released their DNA and citrullinated histone H3 to form NETs upon stimulation with PMA (Fig. 1f,g) (The videos of NETosis can be found as Supplementary videos online). We found that NETosis happened when human neutrophils were treated with either 20 nM or 100 nM PMA for either two hours or overnight (Supplementary Fig. S1 online). Controls for immunofluorescence are shown in Supplementary Fig. S2 online.

### Role of neutrophils and NETs in clot formation

**Figure 2:**
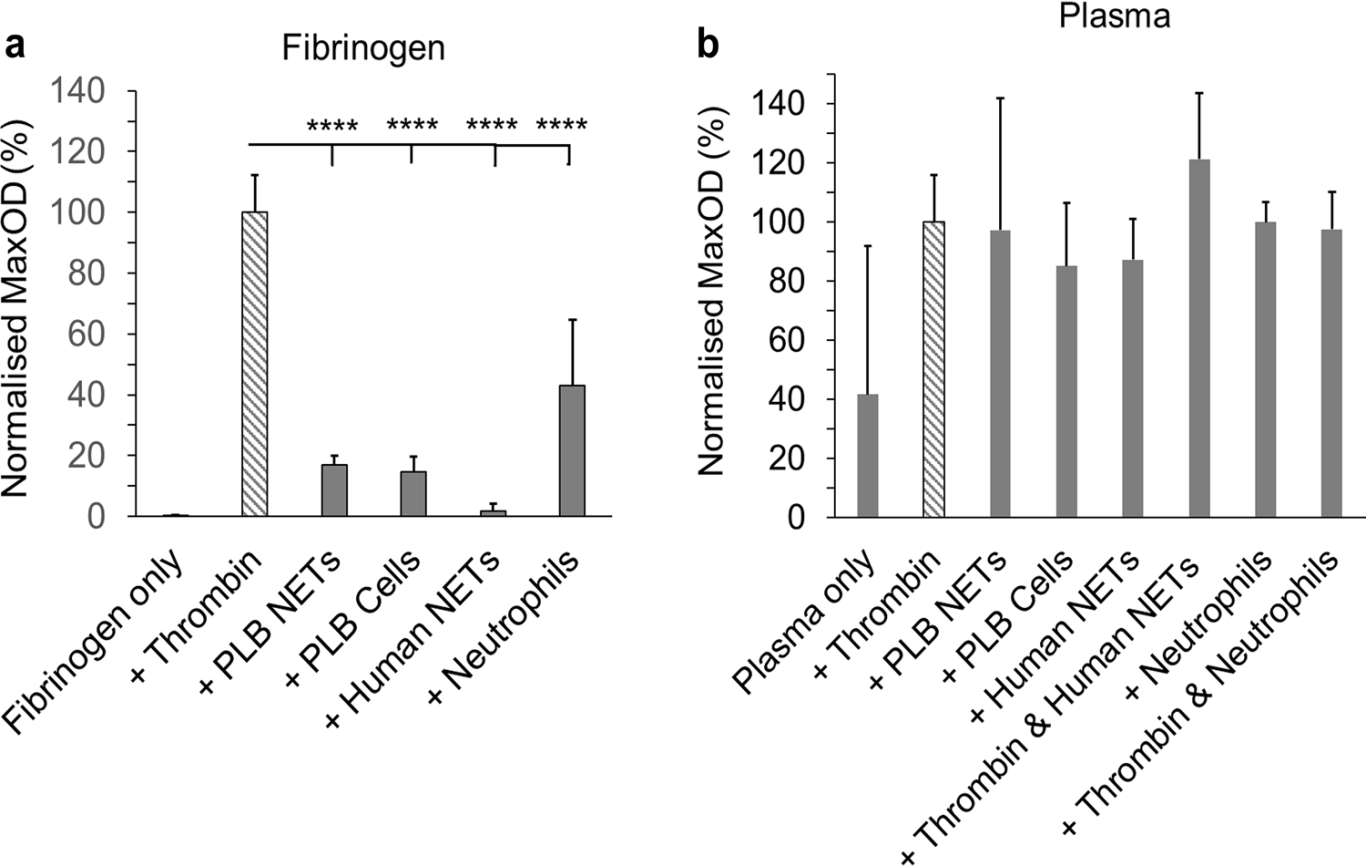
Effects of neutrophils and NETs on coagulation. Turbidity measurements were carried out in (**a**) a purified fibrinogen system or in (**b**) NPP. Differentiated PLB-985 cells or human neutrophils (200,000 cells/100 µl) or NETs (generated from 200,000 cells/100 µl) were added to purified fibrinogen or plasma. Normalized percentage of MaxOD (compared to thrombin only controls) was quantified. Other concentrations: thrombin (0.1 U/ml), fibrinogen (2 mg/ml), plasma (diluted 1:6) and CaCl_2_ (1.5 mM in purified fibrinogen or 3.33 mM in plasma). Error bars represent SD of three technical replicates in triplicates. ****P < 0.0001.

Turbidity measurements were carried out to investigate the role of neutrophils or NETs in clot formation. In a purified fibrinogen system (Fig. 2a), both neutrophils and NETs did not trigger noteworthy fibrin polymerisation, when compared to the positive control (clotting triggered by thrombin), as evidenced by the low maximum absorbance. However, in plasma, clot formation was observed in samples containing neutrophils or NETs, independent of thrombin (Fig. 2b). The lag time of clotting triggered by human NETs in plasma was at least fourfold (*P* < 0.0001) greater than thrombin only controls (Supplementary Fig. S3 online).

**Figure 3:**
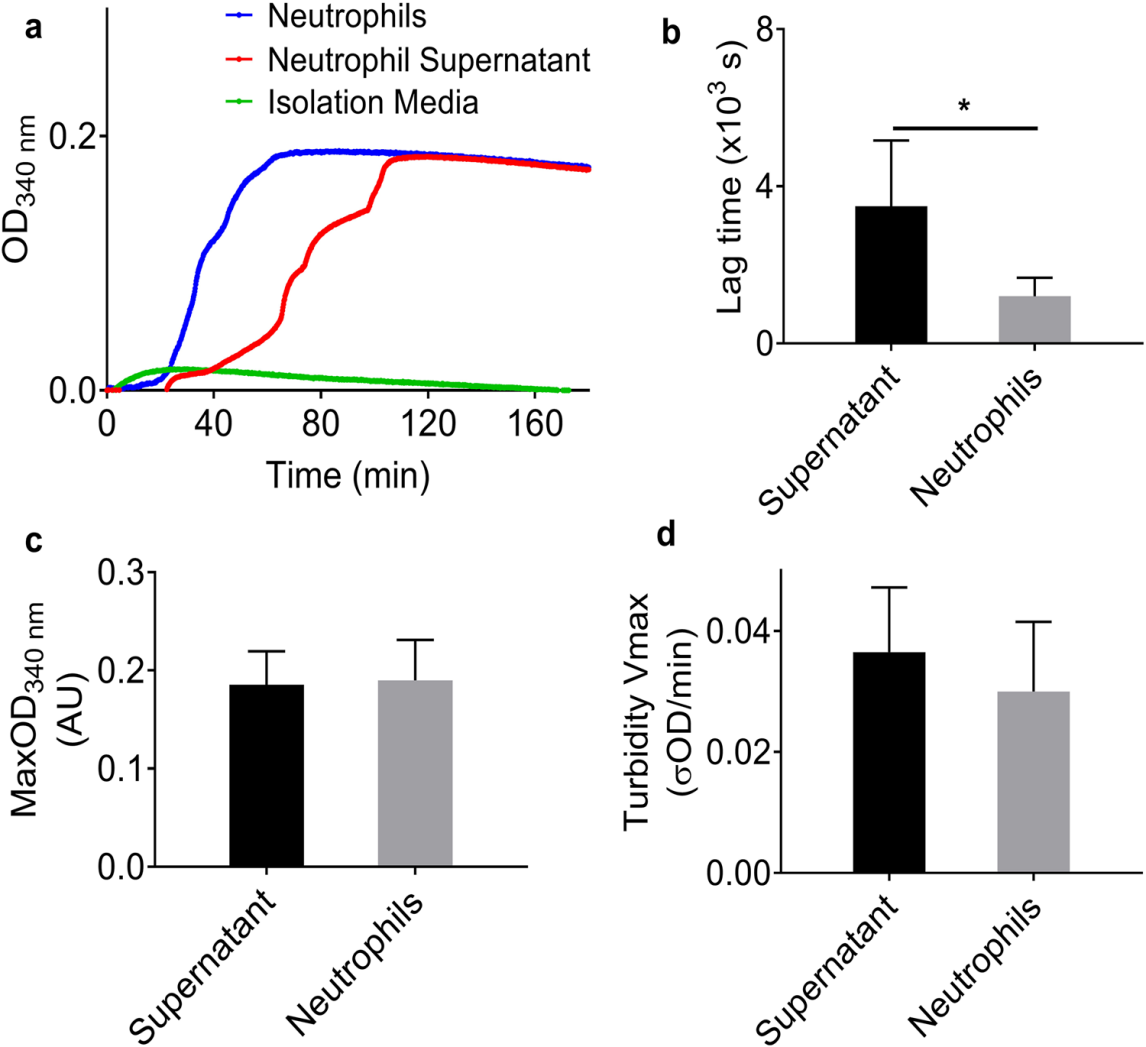
Effects of human neutrophil supernatant on clot formation. (**a**) Clot polymerization curves, obtained by turbidity, showing the effects of human neutrophils (200,000 cells/100 µl) and their supernatant on clot formation when added to NPP. (**b**) Lag time, (**c**) MaxOD and (**d**) maximum rate of clot formation (turbidity Vmax) were quantified. Other concentrations: plasma (diluted 1:6) and CaCl_2_ (3.33 mM). Error bars represent SD of 5–6 replicates. *P < 0.05.

In order to determine if proteins secreted by neutrophils could promote clotting, the supernatant of human neutrophils was collected, added to normal pooled plasma (NPP) and clot formation was investigated by turbidity. Figure 3 shows that the supernatant of neutrophils also promoted clotting, with a slightly longer lag phase and a similar final maximum absorbance as clotting initiated by neutrophils themselves. We next analysed the neutrophil supernatant by mass spectrometry, which indicated that the neutrophils supernatant contained protein S100-A8, cDNA, glyceraldehyde-3-phosphate dehydrogenase, annexin, phosphoglycerate kinase 1, myeloperoxidase (MPO), neutrophil defensin 1 and other proteins (Supplementary Table S1 online).

### Role of neutrophils or NETs in clot dissolution

**Figure 4:**
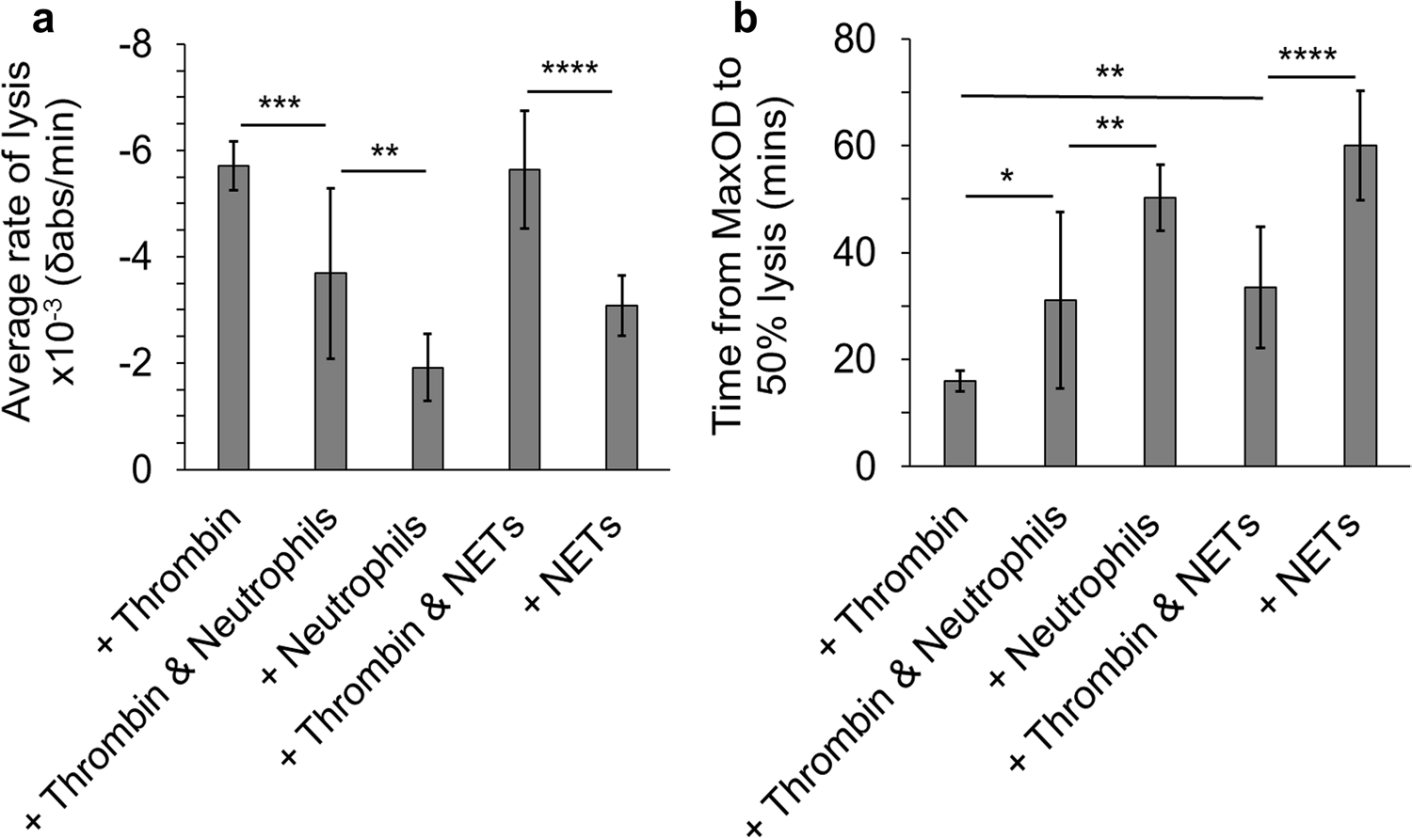
Lysis analysis of the effect of human neutrophils or human NETs on clot dissolution. Neutrophils (200,000 cells/100 µl) or NETs (generated from 200,000 cells/100 µl) were added to plasma. (**a**) Average rate of lysis and (**b**) time from MaxOD to 50% lysis were quantified. Other concentrations: plasma (diluted 1:6), CaCl_2_ (3.33 mM) and thrombin (0.1 U/ml), Error bars represent ± SD of three technical replicates in triplicates. *P < 0.05, **P < 0.01, ***P < 0.001, ****P < 0.0001.

Lysis analysis was next carried out to investigate the role of human neutrophils or NETs in clot dissolution. Clots in the presence of human neutrophils had significantly (*P* < 0.001) lower average rate of lysis than thrombin only controls (Fig. 4a) and took significantly (*P* < 0.05) longer to reach 50% lysis when compared with controls (Fig. 4b). These effects were observed both with and without the addition of thrombin alongside neutrophils. NETs-induced clots had significantly (*P* < 0.0001) lower average rate of lysis than thrombin only controls, but when thrombin was added with NETs, clots had a similar average rate of lysis compared to controls (Fig. 4a). Clots in the presence of NETs took significantly (*P* < 0.01) longer time than controls to reach 50% lysis (Fig. 4b). We observed more pronounced effects of NETs on lysis in the absence of thrombin (*P* < 0.0001).

### Factors involved in the procoagulant effects of neutrophils and NETs

To investigate through which pathway neutrophils/NETs promote clotting, tissue factor (TF) and FXII Inhibitors (TF antibody and Corn trypsin inhibitor (CTI)) were used to block TF and FXII in turbidity. Nevertheless, we saw no effect of these inhibitors in plasma-based experiments (Supplementary Fig. S4 online). Next, FXII-, FXI- and FVII-deficient plasmas were used in turbidity to investigate the procoagulant effects of human neutrophils and NETs.

**Figure 5:**
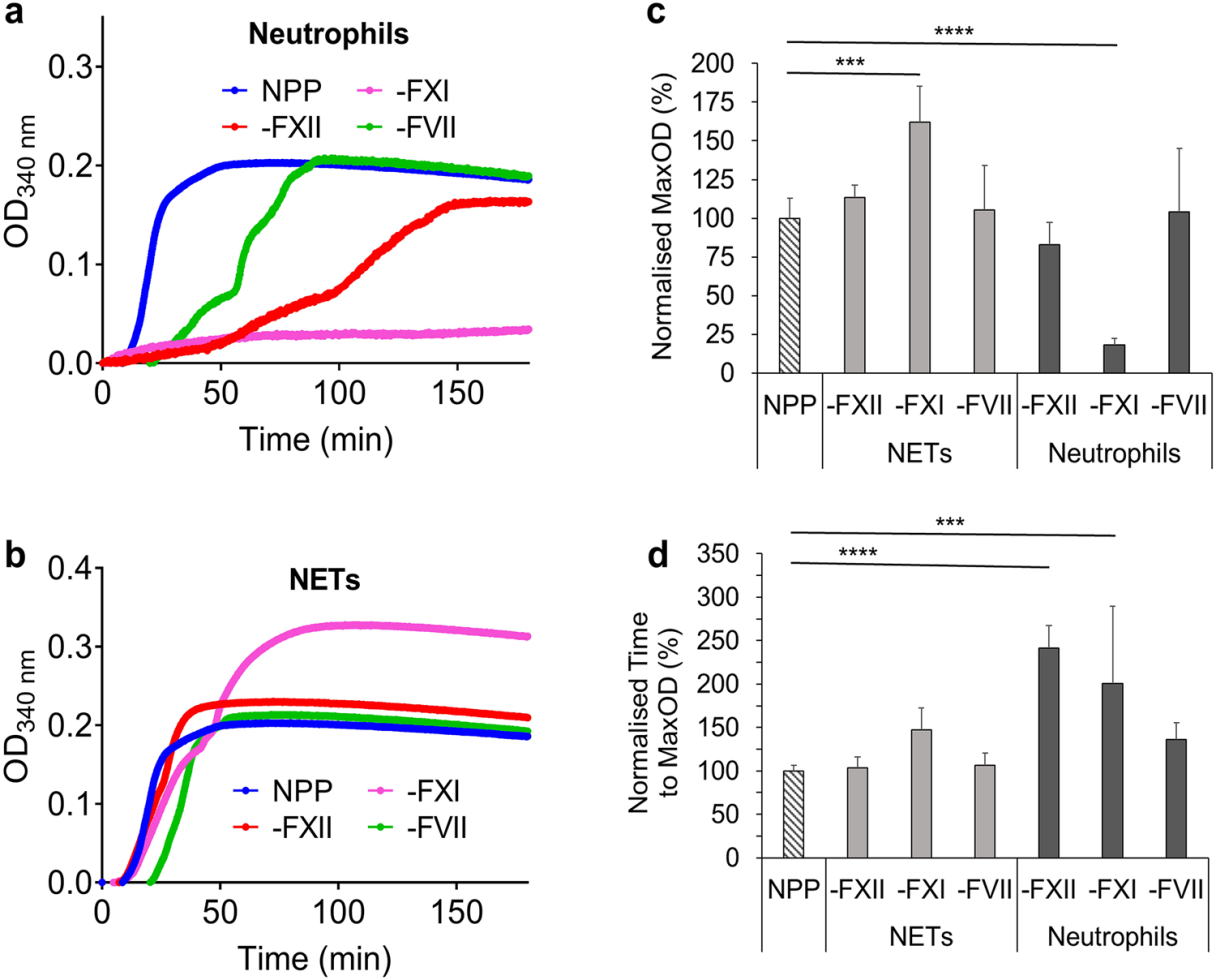
Turbidity analysis of the effect of human neutrophils and human NETs on FXII, FXI and FVII deficient plasmas. (**a**) Human neutrophils (200,000 cells/100 µl) or (**b**) human NETs (generated from 200,000 cells/100 µl) were added into FXII, FXI or FVII deficient plasmas, NPP was used as the positive control. (**c**) Normalized percentage of MaxOD and (**d**) normalized percentage of time to MaxOD were quantified. Other concentrations: plasmas (diluted 1:6) and CaCl_2_ (3.33 mM). Error bars represent SD. NPP data represents 8 replicates, deficient plasma data represents 4–6 replicates. ***P < 0.001, ****P < 0.0001.

Procoagulant effects of neutrophils were still observed in FXII- and FVII-deficient plasmas (Fig. 5a), while procoagulant effects of NETs were still observed in all of these deficient plasmas (Fig. 5b). The time to MaxOD of neutrophil-induced clotting was significantly lengthened in FXII- (*P* < 0.0001) and FXI-deficient (*P* < 0.001) plasmas (Fig. 5d). In FXI-deficient plasma, NETs induced clotting with a significantly (*P* < 0.001) higher MaxOD than NPP controls, but samples containing neutrophils only had significantly (*P* < 0.0001) lower MaxOD than NPP controls (Fig. 5c).

### Role of neutrophils and NETs in clot structure and permeability

**Figure 6:**
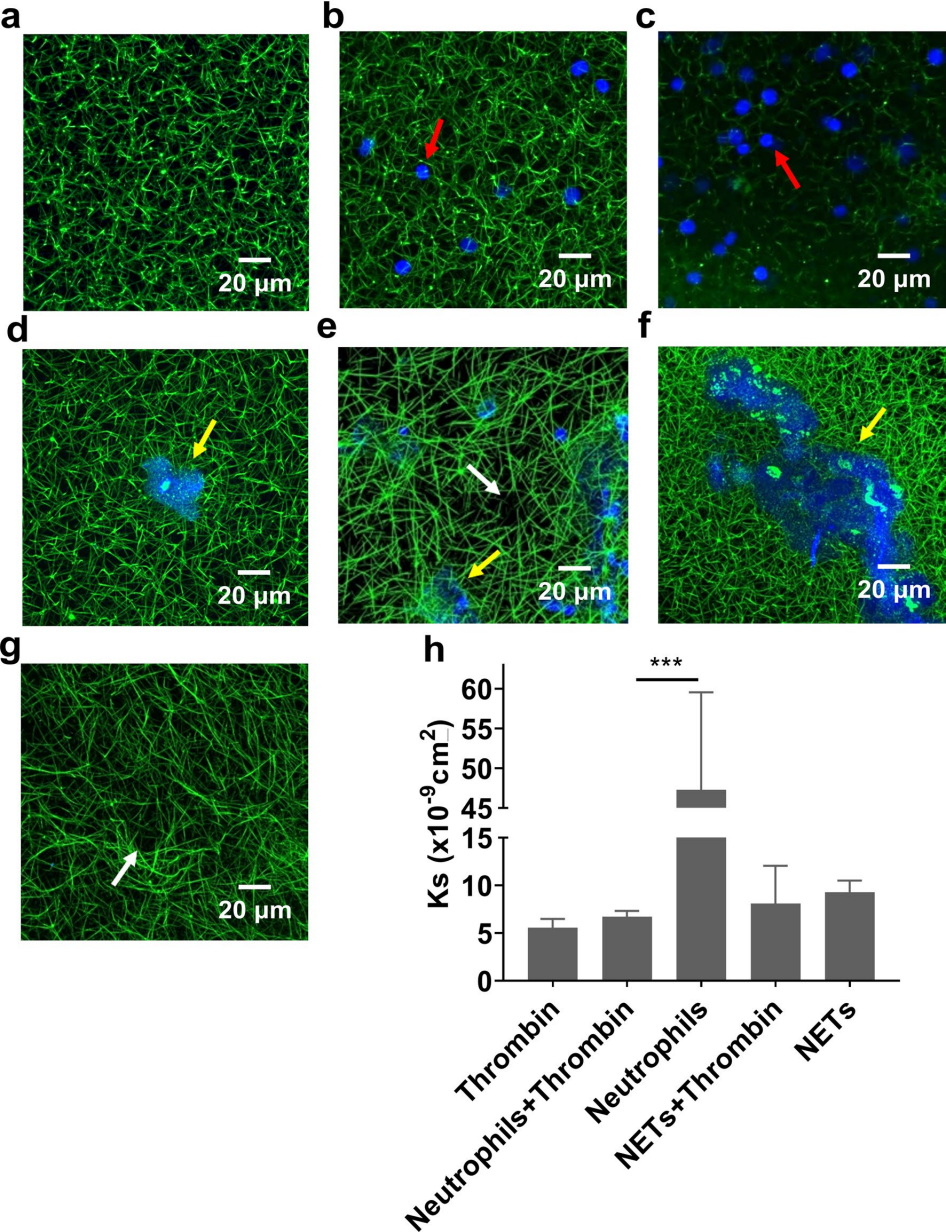
Clot density and the permeability of clots with human neutrophils or human NETs. (**a**) The overall structure of NPP clots triggered with only thrombin, (**b**) thrombin and neutrophils (200,000 cells/100 µl), (**c**) neutrophils only, (**d** and **e**) thrombin and NETs (generated from 200,000 cells/100 µl) and (**f** and **g**) NETs only were visualized by an confocal microscopy with a 40× oil immersion objective lens (1.4 NA). **f** and **g** are images of two positions from the same slide. Blue: DAPI-stained DNA. Green: Alexa Fluor 488 labeled fibrinogen. Red arrows indicate neutrophils, yellow arrows indicate NETs and white arrows indicate large pores. (**h**) The permeation coefficient (Darcy constant [Ks]) of clots were quantified. Other concentrations: plasma was diluted 1:6 for confocal (except in figure **e** was 1:3) and 1:3 for permeation, CaCl_2_ (3.33 mM), thrombin (0.1U/ml). Error bars represent SEM of three technical replicates in triplicates. ***P < 0.001.

Confocal microscopy was used to investigate the effect of human neutrophils and NETs on the overall fibrin fibre network structure of clots. Neutrophils did not visually alter the clot density in the presence of thrombin (Fig. 6b). Unlike the turbidity data, the procoagulant effects of neutrophils were not obvious in the absence of thrombin (Fig. 6c). On the other hand, NETs visually triggered clotting (Fig. 6d,f). Compared to thrombin only controls (Fig. 6a), NETs increased the density of clots, particularly in the areas immediately surrounding the NETs (Fig. 6e,f). In areas where no NETs were formed, we observed large pores (Fig. 6e,g). Permeation experiments were carried out to investigate the permeability of clots. Neutrophil-induced clots were weak and easily ruptured during experiments, showing a significantly (*P* < 0.001) higher permeation coefficient than other conditions (Fig. 6h). When thrombin was added to neutrophils, the clot permeability matched that of control clots. NETs did not significantly affect the permeation coefficient of clots (Fig. 6h). NETs-induced clot permeability and stability were independent of thrombin.

## Discussion

The role of neutrophils and NETs in coagulation in vivo has been investigated in several previous studies. Puhr–Westerheide and colleagues found that neutrophils contribute to platelet aggregation, thereby promoting clotting in the microvasculature^38^. However, Rumbaut and colleagues suggested that microvascular clotting could be neutrophil-independent in the presence of lipopolysaccharide (LPS)^39^. In addition, NETs have been shown to play a crucial role in coagulation in lower-shear vessels, but some studies show that they may be dispensable in those with high-shear^30,38,40^. The procoagulant effects of neutrophils and NETs in vivo are complex and varied. In this in vitro study, we investigated the mechanisms of how neutrophils and NETs interact with fibrin(ogen) in blood clotting using both differentiated PLB-985 cells and isolated human neutrophils. According to our flow cytometry results, 93.6% of isolated cells from human neutrophils expressed both CD16 and CD66b markers. CD66b, a specific marker for secondary and tertiary granules^41,42^, is mainly expressed on neutrophils and eosinophils^43^. CD16 is also expressed on neutrophils, natural killer cells and macrophages, but not on eosinophils^44^. Therefore, our data indicate that highly pure neutrophils were harvested after isolation from human blood.

The expression of neutrophil cell-surface markers (CD16, CD66b and CD11b) on differentiated PLB-985 cells was also evaluated using flow cytometry. CD11b is a marker for secondary, tertiary granules and secretory vesicles^45^, it also plays an important role in adhesion, migration and degranulation of neutrophils^46,47^. Although the expression of CD16 on PLB-985 cells significantly increased upon stimulation with 1.25% Dimethyl sulfoxide (DMSO), expression was still much weaker than that of human neutrophils. Additionally, both normal and differentiated PLB-985 cells had low CD66b expression. However, the expression of CD11b on PLB-985 cells dramatically increased upon differentiation by DMSO to a similar level to that of human neutrophils. Citrullinated histone H3 (H3Cit) plays an important role in the decondensation of chromatin, as the release of decondensed chromatin is an essential process of NETosis, H3Cit has recently been considered as a biomarker of NETs formation^48^. Our immunofluorescence results using a green dye marker for histone H3 show that both human neutrophils and differentiated PLB-985 cells successfully formed NETs which are positive for H3Cit. These findings suggest that even though DMSO-differentiated PLB-985 cells did not possess the same granules as human neutrophils, the “NETosis” capabilities of these differentiated cells are similar to that of human neutrophils. Our data altogether show that PLB-985 cells are different from neutrophils and their extracellular traps may therefore not be fully identical to NETs. Nevertheless, experiments using this cell line were conducted prior to using human neutrophils in order to set up methods as well as prevent unnecessary blood draw. However, since this cell line cannot fully differentiate into primary neutrophils, it could not be a substitute for mature neutrophils, and human neutrophils were always needed to confirm experimental results.

Our turbidity results show that differentiated PLB cells, human neutrophils and their NETs had no significant influence on clot formation in a purified fibrinogen system in the absence of thrombin. However, in plasma they distinctly promoted clotting independently of thrombin. Previous studies showed that neutrophils contribute to clot formation via NETs^6,27,40^. To investigate the potential mechanism of neutrophil-induced clotting, we analysed neutrophil supernatant by mass spectrometry and used it in turbidity experiments. Several mediators were observed in the neutrophil supernatant, including MPO, a marker of neutrophil activation^49^. Although cDNA was also observed in the supernatant, no histones (e.g., H3Cit) were detected. Therefore, these findings suggest that neutrophils were potentially activated to release proteins/enzymes and DNA, but did not form NETs during isolation. We also found that neutrophil-induced clotting had a significantly shorter lag time than both supernatant-induced and NETs-induced clotting, but with fluctuating turbidity curves for both neutrophil-induced and supernatant-induced clots, particularly before reaching MaxOD. This may indicate that, after isolation, neutrophils were still undergoing further activation during the pre-coagulation period. The procoagulant effects we observed in neutrophil experiments may be due to mediators released from activated neutrophils. Confocal images show that NETs increased the local clot density, while neutrophils-induced clots were less stable. Altogether, our results show that neutrophils and NETs have different effects on clot formation, despite the NETs being generated from neutrophils.

A 1985 study by Griep *et.al.*, indicated that negatively charged surfaces (e.g. DNA) can activate FXII^50^. A more recent study has shown that NETs promote coagulation via the intrinsic pathway, where the authors observed reduced thrombus formation in mice injected with the FXII inhibitor PCK (not depicted) or in FXII- and FXI-deficient mice^40^. In contrast, our results suggest that the procoagulant effects of NETs are not mediated through FVII, FXII or FXI but likely involve other components present in the plasma. We observed that FXII- and FXI-deficient plasmas reduced the procoagulant effects of partially activated neutrophils via lengthening the time to the maximum absorbance. Our results with deficient plasmas also highly suggest that partially activated neutrophils or mediators they release could induce clotting via FXI. However, there are currently no studies showing that the proteins we detected by mass spectrometry have direct interactions with FXI. Interestingly, anticoagulant protein annexin, which has been shown to inhibit the activation of factor XII^51^, was observed in the neutrophils’ supernatant. However, it has no apparent inhibitory effect on coagulation in plasmas containing FXII.

Previous studies have shown that NETs increase the fiber thickness and strengthen the clot structure, resulting in decreased permeability and delayed clot lysis^31,52,53^. In this study, we confirmed that both partially activated neutrophils and NETs delayed clot lysis to varying degrees. However, our permeation data show that there were no significant differences in permeability between NET-induced clots and thrombin-induced clots, suggesting their average pore sizes are similar. This may be due to the fact that NETs only increased the density of fibrin fibers in areas directly surrounding them, whereas away from NETs, large pores were visible, indicating that the overall permeability of clots was not altered. Moreover, this finding indicates that the procoagulant effects of NETs could be concentration dependent, since we observed a denser clot structure in areas with more NETs.

To conclude, our data show that partially activated neutrophils and NETs independently promote clotting in plasma via novel mechanisms in vitro, and that FXI and to a lesser degree FXII play a role in the procoagulant effects of neutrophils but not NETs. We also find that partially activated neutrophils and NETs delay clot lysis and that clot density is visually increased in the immediate areas surrounding NETs. We propose that the procoagulant effects of neutrophils could be induced by neutrophil-secreted proteins. These findings provide unique mechanistic insights into the cross-talk between neutrophils, NETs and blood coagulation, with important implications for diseases of thrombosis such as stroke, deep vein thrombosis and heart attacks. Targeting mechanisms involved in this cross-talk provide tempting new prospects for future therapeutics.

## Methods

### Human neutrophils isolation

Whole blood samples were obtained from the antecubital vein of healthy volunteers with minimal stasis, discarding the first 2.5 ml, and collected on 0.5 M Ethylenediaminetetraacetic acid (EDTA) or using EDTA Vacutainers. Normally, 5 to 10 ml of blood were needed for carrying out one subsequent experiment. Human neutrophils were isolated as previously described^54^. All reagents that could be filtered were filtered with a 0.2 μm filter prior to use. First, 5 ml of whole blood was carefully layered on the top of 5 ml Lympholyte-poly (Cedarlane). Samples were centrifuged at 500 RCF, 23 °C for 35 min without brakes. Blood was separated into six clear bands, and the layer of neutrophils and all of the isolation media below this layer were carefully transferred into a new Falcon tubes. Cells were then washed once with Hanks' Balanced Salt solution (HBSS) (without Ca^2+^/Mg^2+^) and centrifuged at 350 RCF for 10 min. After centrifugation, the supernatant was removed, then 2 ml of Red Blood Cell (RBC) Lysis Buffer (Roche) was added to the tube to lyse the residual RBC, and the cell pellet was gently resuspended. Cells were then washed once with 10 ml HBSS (without Ca^2+^/Mg^2+^) and centrifuged at 250 RCF for 5 min. Finally, the supernatant was discarded and the pellet was resuspended in 4 ml of HBSS (with Ca^2+^/Mg^2+^) or HEPES-buffered saline (HBS) ((If required for subsequent experiments, these buffers could be supplemented with 2% w/v human serum albumin (HSA) or 2% v/v fetal bovine serum (FBS)). All Blood donors provided informed written consent according to the declaration of Helsinki, and this study was approved by the University of Leeds Medicine and Health Faculty Research Ethics Committee, reference number HSLTLM12045.

### PLB-985 cell culture and differentiation

The PLB-985 cell line (ACC-139), established from human acute myeloid leukemia and previously reported as a suitable neutrophil-like cell model^42^, was purchased from DSMZ. PLB-985 cells were cultured and differentiated as previously described^42^. In short, cells were cultured in RPMI 1640 medium (R8758, Sigma-Aldrich) supplemented with 10% heat-inactivated FBS at 37 °C in a 5% CO_2_ with air humidified incubator. Medium was renewed every 2 days. For differentiation, cells were treated in RPMI 1640 medium supplemented with 1.25% Dimethyl Sulfoxide (DMSO, Sigma-Aldrich) and heat-inactivated FBS at 37 °C in 5% CO_2_ for 5 days. This treatment medium was renewed on day 3. The treated cells were used in experiments on day 6.

### NETs generation

For immunofluorescence, round cover-slips that were pre-coated with poly-l-lysine (0.01% solution, Sigma-Aldrich) were placed in wells of a 24-well cell culture plate, then isolated neutrophils or PLB-985 cells were seeded (~ 200,000 cells) in 500 μl RPMI 1640 medium (supplemented with 2% FBS) per well and incubated at 37 °C in a 5% CO_2_. After 1 h, the supernatant was gently removed, after which 100 nM PMA (Sigma-Aldrich), which diluted in RPMI 1640 or HBS, were added in each well. Cells were incubated at 37 °C in a 5% CO_2_ incubator overnight (minimally 2 h). All reagents that could be filtered were filtered with a 0.2 μm filter prior to use.

For turbidity assays, cells were treated with 20 nM PMA in a T25 flask, incubated at 37 °C in 5% CO_2_ for 4 h prior to experiments. After treatment, samples were centrifuged at 500 RCF for 5 min to precipitate residual cells or debris, then the supernatant was centrifuged at 17,000 RCF for 15 min at 4 °C to harvest NETs. NETs were washed once with HBS by centrifugation at 17,000 RCF for 15 min at 4 °C, then the supernatant was discarded, and NETs were resuspended in HBS. Before resuspended NETs were used in experiments, double-stranded DNA (dsDNA) was quantified using LabTech-Nanodrop ND100 spectrophotometer at a wavelength of 260 nm to confirm that the NET concentration was consistent in every experiment. All reagents that could be filtered were filtered with a 0.2 μm filter prior to use.

### Turbidity measurements

Turbidity measurements were used to analyse the kinetics of clot formation and fibrinolysis as previously described^55,56^. Final concentrations were as follows: *Fibrinogen System:* 2 mg/ml Human Fibrinogen (von Willebrand factor and plasminogen depleted, Enzyme Research Laboratories), 1.5 mM CaCl_2_ and 200,000 cells or NETs (pre-generated from 200,000 cells) were diluted in HBS and premixed in a 96-well plate. 0.1U/ml thrombin (Merck) was added to initiate clotting. The absorbency was read at 340 nm, every 12 s for 4 h at 37 °C by Multiskan FC Microplate Photometer (Thermo Fisher Scientific) or a PowerWave HT Microplate Spectrophotometer (BioTek). For lysis, tPA (0.03 mg/μl) was added while other conditions remained unchanged. All experiments were performed in triplicate. *Plasma System:* Plasma (diluted 1:6), 3.33 mM Ca^2+^ and 200,000 cells or NETs (pre-generated from 200,000 cells) were diluted in HBS and premixed in a 96-well plate. 0.1 U/ml thrombin was added to initiate clotting. The absorbency was read at 340 nm, every 12 s for 4 h at 37 °C by Multiskan FC Microplate Photometer. For lysis, tPA (0.03 mg/μl) was added and all other conditions remained unchanged. FXII-, FXI- and FVII-deficient plasmas were purchased from George King Bio-Medical, Inc., USA. All experiments were performed in triplicate. All reagents that could be filtered were filtered with a 0.2 μm filter prior to use.

### Immunofluorescence & fluorescence microscopy

Immunofluorescence was carried out to visualize NETs, which were generated on round coverslips as described above. Anti-Histone H3 (citrulline R2 + R8 + R17) antibody (ab5103) and Goat anti-Rabbit IgG H&L (Alexa Fluor 488) (ab150077) were obtained from Abcam. All reagents that could be filtered were filtered with a 0.2 μm filter prior to use. Samples on 24-well plates were fixed with paraformaldehyde (PFA, 4% in PBS, Sigma-Aldrich) for 30 min inside a fume hood at room temperature, followed by washing 3× with 1 ml PBS per well. Then, samples were incubated in 0.5% Triton X-100 (diluted in PBS) for 1 min at room temperature. After washing 3× with PBS, samples were incubated in blocking buffer (1% w/v bovine serum albumin (BSA), 22.52 mg/ml glycine in PBST (PBS with 0.1% v/v Tween 20)) for 30 min at room temperature to block non-specific binding of the antibodies. Following one washing step with 1% BSA in PBST, samples were incubated in 500 μl primary antibody (anti-Histone H3, 1:250 diluted in 1% BSA) per well overnight at 4 °C. The following day, coverslips were washed 3× with PBS, then incubated with secondary antibody (Goat Anti-Rabbit IgG H&L (Alexa Fluor 488), 1:500 diluted in 1% BSA) for 1 h at room temperature in the dark. Coverslips were then washed 3× with PBS, followed by incubating with 300 nM 4′,6-Diamidino-2-Phenylindole, Dihydrochloride (DAPI, Sigma-Aldrich) for 1–5 min in the dark. After washing twice with distilled water, coverslips were mounted upside down onto glass slides with antifade mounting medium (Vector Laboratories). Slides were allowed to dry for at least 1 h at room temperature in the dark. Slides were imaged via 20x (0.8 NA) or 40x (1.0 NA) lenses with Diode 405 nm laser and Argon 488 nm laser filter by using an Airyscan Upright Confocal Microscope (Zeiss LSM880) or Fluorescence Microscope (Zeiss AX10—Zen software).

Fluorescent Labeling was carried out to visualize clots. FITC labeled fibrinogen (25 μg/ml) was added into plasma. All reagents that could be filtered were filtered with a 0.2 μm filter prior to use. The reaction mixture was prepared by diluting plasmas (diluted 1:6 or 1:3), CaCl_2_ (3.33 mM) and 200,000 cells (with 100 nM PMA) or NETs (pre-generated from 200,000 cells) in HBS. Thrombin (0.1U/ml) was added to initiate clotting. Then 30 μl of the mixture was immediately transferred into a well of an uncoated 8-well Ibidi slide (Ibidi GmbH). The slide was placed into a dark humidity chamber for 2 h at room temperature. Slides were imaged via 40× oil immersion objective lens (1.4 NA) with Diode 405 nm laser and Argon 488 nm laser filter by using an Airyscan Inverted Confocal Microscope (Zeiss LSM880). Optical Z-stacks (9.4–10 μm, 21–23 slices) were combined to form 3D images via Fiji-ImageJ.

### Clot permeation

Permeability of clots was analysed as previously described. All reagents that could be filtered were filtered with a 0.2 μm filter prior to use. Clot samples were prepared using similar methods as described for confocal microscopy above, with the following alterations: plasma was diluted 1:3 and the 6-channel Ibidi slide was used. Clotting slides were placed horizontally in the moist chamber for 2 h then a syringe was connected to each channel. Syringes were filled with permeation buffer to a set height (4 cm) and clots were washed in this manner for 90–120 min. Flow-through of buffer was subsequently measured by collecting and weighing the buffer flow-through every 5 min for 20–40 min. Volume of buffer flow-through, correlated to weight assuming 1 g = 1 mL, over time was plotted and fitted by linear regression (R^2^ = 0.99). The permeation coefficient (Ks, Darcy constant), corresponding to pore size, was calculated as previously described^57^.

### Statistical analysis

All statistical analyses were performed using GraphPad Prism 7. A normal distribution of the data used in two-tailed unpaired t-test was checked by Shapiro–Wilk (W) test (*P* > 0.05). Differences in the expression levels of CD16 (W = 0.9306) and CD66b (W = 0.9355) on differentiated PLB-985 cells were determined by two-tailed unpaired t-test. Differences in the lag time (W = 0.9445), maximum optical density (MaxOD) (W = 0.9713) and turbidity Vmax (W = 0.9130) of human neutrophil supernatant were determined by two-tailed unpaired t test. Other turbidity and lysis data were determined by one-way ANOVA analysis with Tukey's multiple comparisons test (Alpha = 0.05). Differences in Ks were determined by one-way ANOVA with Tukey's multiple comparisons test (Alpha = 0.05). Contrast of confocal and fluorescence images were adjusted by Fiji-Image J. *P* values < 0.05 were considered to indicate statistical significance. All methods and analyses were performed in accordance with the relevant guidelines and regulations.

## Supplementary Information

Supplementary Video 1.

Supplementary Video 2.

Supplementary Information 1.

## Data Availability

The datasets generated during and/or analysed during the current study are available from the corresponding author on reasonable request.
