# Supplementary material for: Neutrophils can promote clotting via FXI and impact clot structure via neutrophil extracellular traps in a distinctive manner in vitro
Source: Sci Rep. 2021 Jan 18;11:1718. doi: 10.1038/s41598-021-81268-7 (PMC7814028; doi:10.1038/s41598-021-81268-7)
Supplement: Supplementary file 3 — Supplementary Information 1. [file 41598_2021_81268_MOESM3_ESM.pdf]

Neutrophils can Promote Clotting via FXI and Impact Clot Structure via Neutrophil Extracellular Traps in a Distinctive Manner *in vitro*

Y. Shi[1], J.S. Gauer[1], S.R. Baker[1,2], H. Philippou[1], S.D. Connell[3], R.A.S. Ariëns[1*]

[1]Discovery and Translational Science Department, Institute of Cardiovascular and Metabolic Medicine, University of Leeds, Leeds, LS2 9LU, United Kingdom.

[2]Department of Physics, Wake Forest University, Winston Salem, NC, United States.

[3]The Astbury Centre for Structural Molecular Biology, Molecular & Nanoscale Physics, University of Leeds, Leeds, LS2 9JT, United Kingdom.

*Address correspondence to Robert A.S. Ariëns, LIGHT Laboratories, University of Leeds, Clarendon Way, Leeds, LS2 9JT, United

Kingdom. Phone: +44 (0) 113 34 3437734; Email: r.a.s.ariens@leeds.ac.uk.

Supplementary Methods

All methods and analyses were performed in accordance with the relevant guidelines and regulations.

Normal Pooled Plasma

Free-flowing blood samples were collected from the antecubital vein of at least 20 healthy donors by using the 19 gauge butterfly needle. Blood samples were taken into 0.109 M trisodium citrate and were used within 1 hour. Samples were centrifuged at 3,000 RCF for 20-30 minutes to obtain platelet poor plasma, and then pooled. Normal pooled plasma (NPP) was divided into 0.8-1 ml aliquots, and snap-frozen in liquid nitrogen, then stored at –80 C. All blood donors provided informed written consent according to the declaration of Helsinki, and this study was approved by the University of Leeds Medicine and Health Faculty Research Ethics Committee, reference number HSLTLM12045.

Human Neutrophils Count and Supernatant Collection

Following the neutrophil isolation, the number of live neutrophils was determined by mixing 10 µl cell suspension and 90 µl trypan blue then transferring 10 ul of mixture onto a hemocytometer. The number of live neutrophils was counted under an Olympus CKX41 inverted microscope.

The neutrophil sample was centrifuged at 500 RCF for 5 minutes to pellet the cells and obtain the supernatant. This neutrophil supernatant was used in turbidity measurements directly. A sample of supernatant was sent to the Biomolecular Mass Spectrometry Facility, Astbury Centre for Structural Molecular Biology, School of Molecular and Cellular Biology, University of Leeds for protein identification by mass spectrometry.

Flow Cytometry

Flow cytometry was used to investigate the purity of isolated human neutrophils and to identify whether PLB-985 cells were successfully differentiated into a functional neutrophil-like phenotype by analysing the expression of human neutrophil surface antigens CD16[45], CD66b[44] and CD11b[46]. PE anti-human CD11b Antibody (clone: RUO), APC/Fire 750 anti-human CD16 Antibody (clone: B73.1) and FITC anti-human CD66b Antibody (clone: G10F5) were purchased from BioLegend. All reagents that could be filtered were filtered with a 0.2 µm filter prior to use. Cells were counted and adjusted to a concentration of 1,000,000 cells/ml in 15 ml Falcon tubes, then washed once with PBS (with 10% v/v FBS) by centrifugation at 250 RCF for 5 minutes. Cells were incubated in Human BD Fc Block (BD Biosciences) (5 µl per 100 µl ice cold PBS) for at least 10 mins. Then, 5 µl primary labelled antibodies were added directly in each tube without washing. After 30 mins incubation at 4°C in the dark, cells were washed 3 times with PBS (10% v/v FBS) by centrifugation at 250 RCF for 5 minutes, after which each pellet was resuspended in 500 µl to 1 ml PBS (10% v/v FBS). Samples were analysed using a CytoFLEX S - 4 laser flow cytometer, where the intensity of forward light scatter channel (FSC) reflected the size and surface area of cells, while the side light scatter channel (SSC) reflected the internal complexity and granularity of the cells[60]. Experiments were performed in triplicate.

Supplementary Figures and Table

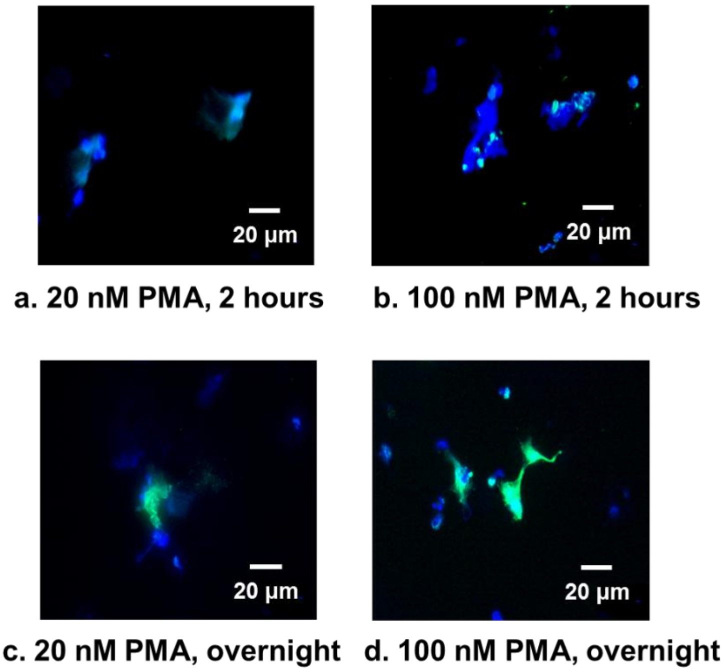

Figure S1. Fluorescence microscopy images of NETs generated from isolated human neutrophils by stimulating with PMA. Blue: DAPI-stained DNA. Green: Alexa Fluor 488 labelled Histone H3.

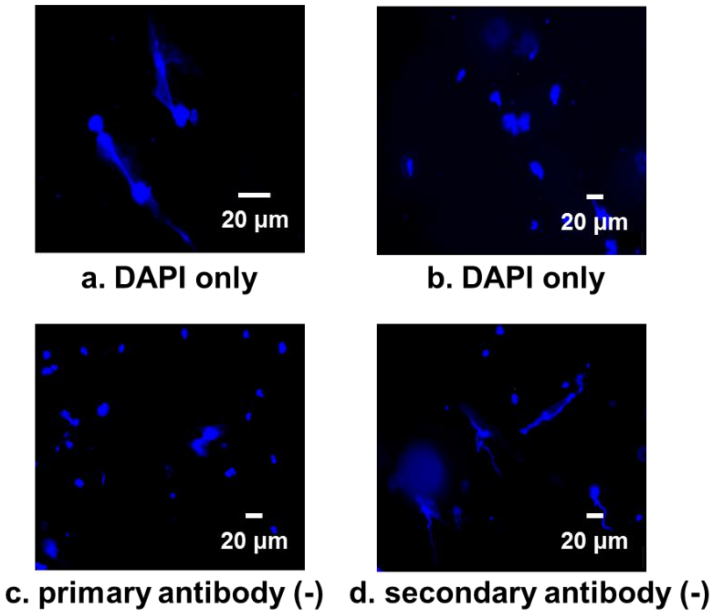

Figure S2. Controls for immunofluorescence. Blue: DAPI-stained DNA. Primary antibody: anti-Histone H3 (citrulline R2 + R8 + R17). Secondary antibody: goat anti-Rabbit IgG H&L (Alexa Fluor 488).

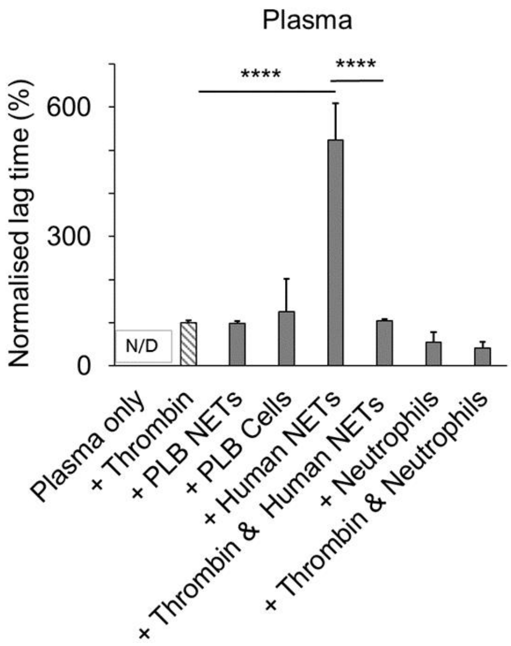

Figure S3. Effects of neutrophils and NETs on coagulation. Turbidity measurements were carried out in NPP. Differentiated PLB-985 cells or human neutrophils (200,000 cells/100 µl) or NETs (generated from 200,000 cells/100 µl) were added to plasma. Normalized percentage of lag time (compared to thrombin only controls) was quantified. Other concentrations: thrombin (0.1 U/ml), plasma (diluted 1:6) and $CaCl_2$ (3.33 mM). Error bars represent SD of three technical replicates in triplicates. **** $P < 0.0001$.

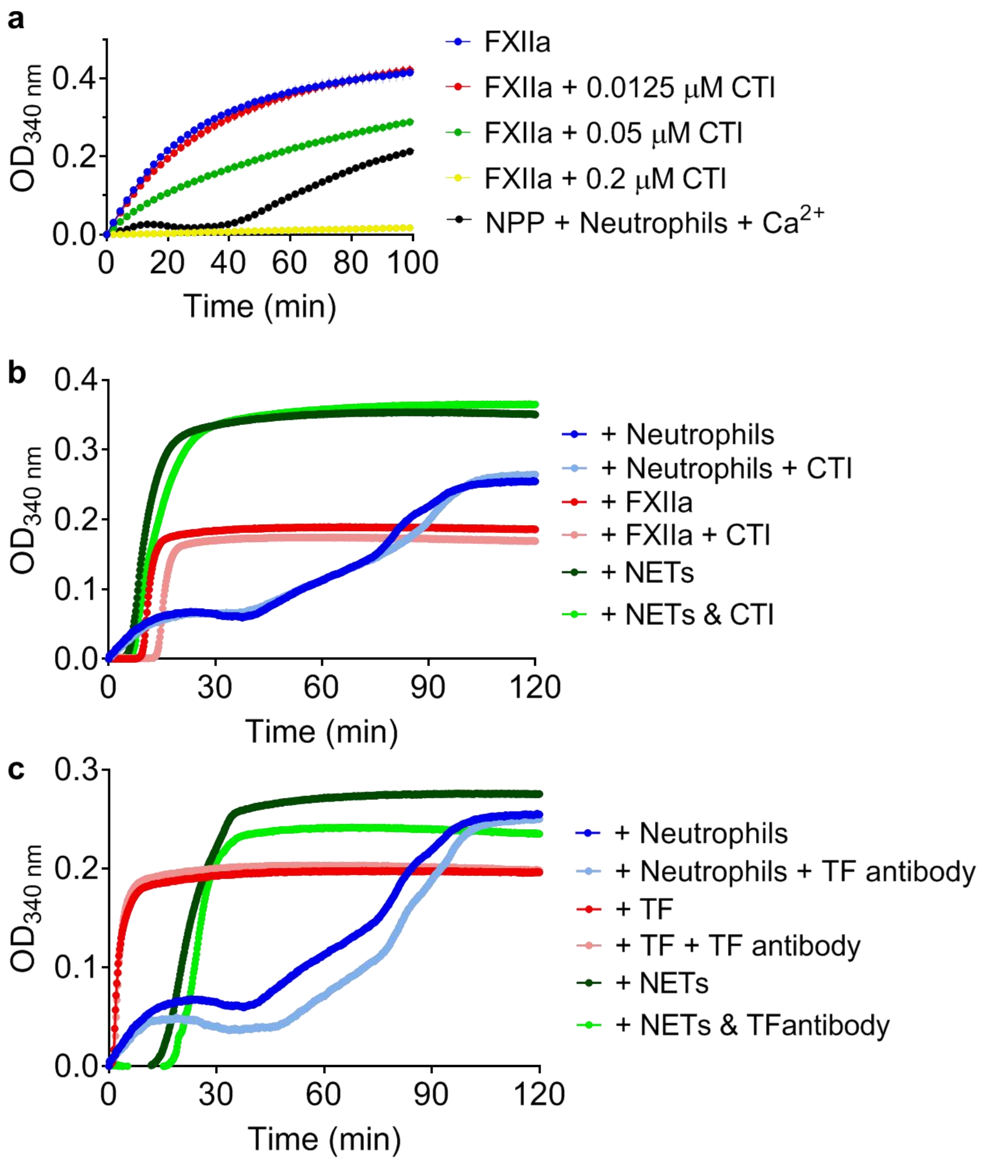

Figure S4. Role of TF and FXII Inhibitors in the procoagulant effects of neutrophils and NETs in plasma. (**a**) Chromogenic Activity Assay was carried out in a purified system with 30 nM FXIIa 0.0125 μM, 0.05 μM or 0.2 μM CTI was added to block FXII. NPP (diluted 1:5), human neutrophils (200,000 cells/100 μl) and 5 mM Ca^{2+} together were added instead of 3 nM FXIIa as a control to show an approximate concentration of FXIIa that may contained in the turbidity plasma system. (**b** and **c**) Turbidity measurements were carried out in NPP. 3.33 mM Ca^{2+} with human neutrophils (200,000 cells/100 μl) or NETs (generated from 200,000 cells/100 μl) or 30 nM FXIIa or TF (diluted 1:30) were added to plasma (diluted 1:6). TF antibody (diluted 1:50) was added to block TF, 1.6 μM CTI was added to block FXII. All data represents >3 replicates.

Table S1. Mass spectrometry data showing the composition of human neutrophil supernatant.

Accession *	Protein Description	-10lgP **
H6VRF8	Keratin 1, GN=KRT1 PE=3 SV=1	176.5
P13645	Keratin type I cytoskeletal 10, GN=KRT10 PE=1 SV=6	155.61
P35908	Keratin type II cytoskeletal 2 epidermal, GN=KRT2 PE=1 SV=2	150.21
P35527	Keratin type I cytoskeletal 9, GN=KRT9 PE=1 SV=3	147.8
P05109	Protein S100-A8, GN=S100A8 PE=1 SV=1	122.94
B7ZAL5	cDNA FLJ79229 highly similar to Lactotransferrin, PE=2 SV=1	120.31
B4DUI5	Triosephosphate isomerase, PE=2 SV=1	109.63
B2R4M6	Protein S100, PE=2 SV=1	103.9
B4DVQ0	cDNA FLJ58286 highly similar to Actin cytoplasmic 2, PE=2 SV=1	103.84
A0A0M4FNU3	Fructose-bisphosphate aldolase (Fragment), GN=ALDOA PE=3 SV=1	91.97
P04406	Glyceraldehyde-3-phosphate dehydrogenase, GN=GAPDH PE=1 SV=3	89.44
P13647	Keratin type II cytoskeletal 5, GN=KRT5 PE=1 SV=3	87.82
B4DJI1	cDNA FLJ52549 highly similar to L-lactate dehydrogenase A chain, PE=2 SV=1	77.88
A0A4D5RAJ5	Annexin, PE=3 SV=1	72.46
P00558	Phosphoglycerate kinase 1, GN=PGK1 PE=1 SV=3	72.41
P05164	Myeloperoxidase, GN=MPO PE=1 SV=1	69.62
B4DEK3	cDNA FLJ56959 highly similar to Vascular endothelial growth factor receptor 2, PE=2 SV=1	69.24
B2R4C5	Lysozyme, GN=LYZ PE=2 SV=1	67.95
A0A0K0K1H9	Epididymis secretory protein Li 48 (Fragment), GN=HEL-S-48 PE=2 SV=1	67.16
K7EPF6	6-phosphogluconate dehydrogenase decarboxylating (Fragment), GN=PGD PE=1 SV=1	63.71
P59665	Neutrophil defensin 1, GN=DEFA1 PE=1 SV=1	61.61
B4E3A8	cDNA FLJ53963 highly similar to Leukocyte elastase inhibitor, PE=2 SV=1	60.26
B7Z4U6	cDNA FLJ55803 highly similar to Gelsolin, PE=2 SV=1	59.14
B4DE36	Glucose-6-phosphate isomerase, PE=2 SV=1	57.29
A0A384NPR0	Epididymis secretory sperm binding protein, PE=2 SV=1	53.55
D6RA82	Annexin, GN=ANXA3 PE=1 SV=1	44.84
A0A0K0K1K8	Enolase 1 (Alpha) isoform CRA_a, GN=ENO1 PE=3 SV=1	44.18
Q59ES1	Leukotriene A4 hydrolase variant (Fragment), PE=2 SV=1	44.14

* Accession: The accession number of the protein as seen in the FASTA database.

** -10lgP: The protein confidence score. The protein matches in the reports are ordered by this score. Proteins with a high -10lgP score have many peptides that have good quality sequences.
